# Supplementary material for: Femoral anterior condyle height decreases as the distal anteroposterior size increases in total knee arthroplasty: A comparative study
Source: PLoS One. 2024 Feb 26;19(2):e0297634. doi: 10.1371/journal.pone.0297634 (PMC10896507; doi:10.1371/journal.pone.0297634)
Supplement: S1 File — (ZIP) [file pone.0297634.s001.zip › Supporting Information/TKA measured data table samples.pdf]

床号：10

中国人膝关节数据库

手术日期：2012年5月31日， 住院号：4649928，“ ”处请打✓选择

患者姓名：[redacted] 男 或 女✓： 年龄：57岁	左膝 或 右膝✓
膝内翻角°✓ 或膝外翻角°：	2.5°
股骨外翻角°：	9°
胫骨后倾角°-前缘法：	12.9°
胫骨后倾角°-中轴法：	11°
外平台切骨厚度（mm）：	8
内平台切骨厚度（mm）：	6.5
外平台前后径（mm）：	41.5
平台中部前后径（mm）：	48
内平台前后径（mm）：	53
平台内外径（mm）：	73
股骨远端内侧切骨厚度(mm)：	9
股骨远端外侧切骨厚度(mm)：	8
股骨前方内侧切骨厚度(mm)：	4
股骨前方外侧切骨厚度(mm)：	5
股骨内后髁切骨厚度（mm）：	13
股骨外后髁切骨厚度（mm）：	8.5
术前测量股骨尺寸：A□，B□，C□，D□，E☑，F□，G□，H□	
术中测量股骨尺寸：A□，B□，C□，D□，E☑，F□，G□，H□	
术前测量胫骨尺寸：1号□，2号□，3号□，4号☑，5号□，6号□，7号□，8号□	
术中测量胫骨尺寸：1号□，2号□，3号□，4号☑，5号□，6号□，7号□，8号□	
聚乙烯垫厚度（mm）：10	
如果置换髌骨做以下记录	
髌骨上下极距离：43(cm)， 内侧头止点距髌骨下支：2（cm）	
髌骨假体直径(mm)：	26
髌骨厚度(mm)：	7.5
髌骨假体厚度(mm)：	21.5
保留髌骨骨质厚度(mm)：	14
切除髌骨骨质厚度(mm)：	7.5

骨质评估： 优 中✓ 差

床号: 21

中国人膝关节数据库

手术日期: 2012 年 5 月 31 日， 住院号: 4636674 ，“ ”处请打✓选择

患者姓名: [redacted] 男✓ 或 女 ： 年龄: 61 岁 左膝 或 右膝✓	
膝内翻角° 或膝外翻角° ：	
股骨外翻角°:	
胫骨后倾角°-前缘法:	
胫骨后倾角°-中轴法:	
外平台切骨厚度（mm）：	11
内平台切骨厚度（mm）：	2
外平台前后径（mm）：	42
平台中部前后径（mm）：	48
内平台前后径（mm）：	50
平台内外径（mm）：	70
股骨远端内侧切骨厚度(mm):	9
股骨远端外侧切骨厚度(mm):	8
股骨前方内侧切骨厚度(mm):	6.5
股骨前方外侧切骨厚度(mm):	9.5
股骨内后髁切骨厚度（mm）：	11
股骨外后髁切骨厚度（mm）：	10
术前测量股骨尺寸: A☐, B☐, C☐, D☐, E☐✓, F☐, G☐, H☐	
术中测量股骨尺寸: A☐, B☐, C☐, D☐✓, E☐, F☐, G☐, H☐	
术前测量胫骨尺寸: 1号☐, 2号☐, 3号☐, 4号☐✓, 5号☐, 6号☐, 7号☐, 8号☐	
术中测量胫骨尺寸: 1号☐, 2号☐, 3号☐✓, 4号☐, 5号☐, 6号☐, 7号☐, 8号☐	
聚乙烯垫厚度（mm）：	10
**如果置换髌骨做以下记录**	
髌骨上下极距离: （cm）， 内侧头止点距髌骨下支: （cm）	
髌骨假体直径(mm):	
髌骨厚度(mm):	
髌骨假体厚度(mm):	
保留髌骨骨质厚度(mm):	
切除髌骨骨质厚度(mm):	

骨质评估: 优 中✓ 差

床号: 11

中国人膝关节数据库

手术日期: 2012 年 6 月 4 日, 住院号: 4160180, "" 处请打√选择

患者姓名: [redacted] 男 或 女√: 年龄: 61 岁 左膝 或 右膝√	
膝内翻角°√ 或膝外翻角°:	10°
股骨外翻角°:	7.6°
胫骨后倾角°-前缘法:	8°
胫骨后倾角°-中轴法:	6°
外平台切骨厚度(mm):	7.5+2
内平台切骨厚度(mm):	5+2
外平台前后径(mm):	30.5
平台中部前后径(mm):	41
内平台前后径(mm):	41
平台内外径(mm):	65.5
股骨远端内侧切骨厚度(mm):	8.5
股骨远端外侧切骨厚度(mm):	9
股骨前方内侧切骨厚度(mm):	6.5
股骨前方外侧切骨厚度(mm):	10
股骨内后髁切骨厚度(mm):	7+4
股骨外后髁切骨厚度(mm):	4+4
术前测量股骨尺寸: A□, B□, C□, D☑, E□, F□, G□, H□	
术中测量股骨尺寸: A□, B□, C☑, D□, E□, F□, G□, H□	
术前测量胫骨尺寸: 1号□, 2号□, 3号☑, 4号□, 5号□, 6号□, 7号□, 8号□	
术中测量胫骨尺寸: 1号□, 2号☑, 3号□, 4号□, 5号□, 6号□, 7号□, 8号□	
聚乙烯垫厚度(mm): 10	

如果置换髌骨做以下记录

髌骨上下极距离: (cm), 内侧头止点距髌骨下支: (cm)	
髌骨假体直径(mm):	
髌骨厚度(mm):	
髌骨假体厚度(mm):	
保留髌骨骨质厚度(mm):	
切除髌骨骨质厚度(mm):	

骨质疏松: 轻 中√ 重

床号：11

中国人膝关节数据库

手术日期：2012 年 6 月 4 日，住院号：460180，“ ”处请打✓选择

患者姓名：[redacted] 男 或 女✓：年龄：61 岁 左膝 或 右膝✓	
膝内翻角°✓ 或膝外翻角°：	10°
股骨外翻角°：	7.6°
胫骨后倾角°-前缘法：	8°
胫骨后倾角°-中轴法：	6°
外平台切骨厚度（mm）：	7.5+2
内平台切骨厚度（mm）：	5+2
外平台前后径（mm）：	30.5
平台中部前后径（mm）：	41
内平台前后径（mm）：	41
平台内外径（mm）：	65.5
股骨远端内侧切骨厚度(mm)：	8.5
股骨远端外侧切骨厚度(mm)：	9
股骨前方内侧切骨厚度(mm)：	6.5
股骨前方外侧切骨厚度(mm)：	10
股骨内后髁切骨厚度（mm）：	7+4
股骨外后髁切骨厚度（mm）：	4+4
术前测量股骨尺寸：A□，B□，C□，D□✓，E□，F□，G□，H□	
术中测量股骨尺寸：A□，B□，C□✓，D□，E□，F□，G□，H□	
术前测量胫骨尺寸：1号□，2号□，3号□✓，4号□，5号□，6号□，7号□，8号□	
术中测量胫骨尺寸：1号□，2号□✓，3号□，4号□，5号□，6号□，7号□，8号□	
聚乙烯垫厚度（mm）：10	

如果置换髌骨做以下记录
髌骨上下极距离：（cm），内侧头止点距髌骨下支：（cm）
髌骨假体直径(mm)：
髌骨厚度(mm)：
髌骨假体厚度(mm)：
保留髌骨骨质厚度(mm)：
切除髌骨骨质厚度(mm)：

骨质评估： 好 中✓ 差

床号：39　　　　　　　　　　　　　　　　　　　　　　术者：

中国人膝关节数据库

手术日期：2012 年 6 月 6 日，　住院号：4451938，"　"处请打√选择

患者姓名：[redacted]　男 或 女√：	年龄：70 岁　左膝 或 右膝√
膝内翻角°√　或膝外翻角°：	10°
股骨外翻角°：	5.6°
胫骨后倾角°-前缘法：	6.5°
胫骨后倾角°-中轴法：	4.5°
外平台切骨厚度（mm）：	12
内平台切骨厚度（mm）：	3
外平台前后径（mm）：	42
平台中部前后径（mm）：	41
内平台前后径（mm）：	50
平台内外径（mm）：	70
股骨远端内侧切骨厚度(mm)：	8
股骨远端外侧切骨厚度(mm)：	6
股骨前方内侧切骨厚度(mm)：	4.5
股骨前方外侧切骨厚度(mm)：	6.5
股骨内后髁切骨厚度（mm）：	8
股骨外后髁切骨厚度（mm）：	7
术前测量股骨尺寸：A□，B□，C□，D□，E√，F□，G□，H□	
术中测量股骨尺寸：A□，B□，C□，D□，E√，F□，G□，H□	
术前测量胫骨尺寸：1号□，2号□，3号√，4号□，5号□，6号□，7号□，8号□	
术中测量胫骨尺寸：1号□，2号□，3号■，4号√□，5号□，6号□，7号□，8号□	
聚乙烯垫厚度（mm）：10	
如果置换髌骨做以下记录	
髌骨上下极距离：47.5（cm），内侧头止点距髌骨下支：22.5（cm）	
髌骨假体直径(mm)：	026
髌骨厚度(mm)：	21.5
髌骨假体厚度(mm)：	7.5
保留髌骨骨质厚度(mm)：	14
切除髌骨骨质厚度(mm)：	~~14~~ 7.5

骨质评估：优　中√　差

床号：11

术者

中国人膝关节数据库

手术日期：2011 年 12 月 13 日，住院号：4624942，“ ”处请打✓选择

患者姓名：[redacted] 男 或 女✓：	年龄：58 岁　左膝✓ 或 右膝
膝内翻角°✓　或膝外翻角°：	10.5°
股骨外翻角°：	6.5°
胫骨后倾角°-前缘法：	13.3°
胫骨后倾角°-中轴法：	11.1°
外平台切骨厚度（mm）：	10.5
内平台切骨厚度（mm）：	2.5
外平台前后径（mm）：	40.5
平台中部前后径（mm）：	44.5
内平台前后径（mm）：	51
平台内外径（mm）：	78
股骨远端内侧切骨厚度(mm)：	8.5
股骨远端外侧切骨厚度(mm)：	8
股骨前方内侧切骨厚度(mm)：	0
股骨前方外侧切骨厚度(mm)：	4.5
股骨内后髁切骨厚度（mm）：	8
股骨外后髁切骨厚度（mm）：	6.5
术前测量股骨尺寸：A□，B□，C□，D□，E☑，F□，G□，H□	
术中测量股骨尺寸：A□，B□，C□，D□，E☑，F□，G□，H□	
术前测量胫骨尺寸：1号□，2号□，3号□，4号☑，5号□，6号□，7号□，8号□	
术中测量胫骨尺寸：1号□，2号□，3号□，4号☑，5号□，6号□，7号□，8号□	
聚乙烯垫厚度（mm）：10	
如果置换髌骨做以下记录	
髌骨上下极距离：46（cm），内侧头止点距髌骨下支：23.5（cm）	
髌骨假体直径(mm)：	26
髌骨厚度(mm)：	22
髌骨假体厚度(mm)：	7.5
保留髌骨骨质厚度(mm)：	14.5
切除髌骨骨质厚度(mm)：	7.5

骨质评估：[illegible]　中　✓差

床号：9

术后

中国人膝关节数据库

手术日期：2011 年 12 月 14 日，　住院号：6628767，“ ”处请打✓选择

患者姓名：[redacted]　男 或 女✓：　年龄：76 岁　左膝✓ 或 右膝	
膝内翻角°✓　或膝外翻角°：	16.2°
股骨外翻角°：	6°
胫骨后倾角°-前缘法：	15°
胫骨后倾角°-中轴法：	13°
外平台切骨厚度（mm）：	10
内平台切骨厚度（mm）：	3
外平台前后径（mm）：	35
平台中部前后径（mm）：	~~[illegible]~~ 40.5
内平台前后径（mm）：	37
平台内外径（mm）：	63.5
股骨远端内侧切骨厚度(mm)：	9
股骨远端外侧切骨厚度(mm)：	11
股骨前方内侧切骨厚度(mm)：	2
股骨前方外侧切骨厚度(mm)：	3.5
股骨内后髁切骨厚度（mm）：	14
股骨外后髁切骨厚度（mm）：	7
术前测量股骨尺寸：A□，B□，C□，D□，E□✓，F□，G□，H□	
术中测量股骨尺寸：A□，B□，C□✓，D□，E□，F□，G□，H□	
术前测量胫骨尺寸：1号□，2号□，3号□✓，4号□，5号□，6号□，7号□，8号□	
术中测量胫骨尺寸：1号□，2号□✓，3号□，4号□，5号□，6号□，7号□，8号□	
聚乙烯垫厚度（mm）：10	
**如果置换髌骨做以下记录**	
髌骨上下极距离：37（cm），　内侧头止点距髌骨下支：13（cm）	
髌骨假体直径(mm)：	
髌骨厚度(mm)：	18.5
髌骨假体厚度(mm)：	
保留髌骨骨质厚度(mm)：	
切除髌骨骨质厚度(mm)：	

骨质评估：　优　✓中　差

床号: 27

术者

中国人膝关节数据库

手术日期: 2011 年 12 月 14 日， 住院号: 4628643，" " 处请打 ✓ 选择

患者姓名: [redacted]　男 或 女 ✓: 年龄: 61 岁	左膝 ✓ 或 右膝
膝内翻角° ✓ 或膝外翻角°:	10°
股骨外翻角°:	5°
胫骨后倾角°-前缘法:	10°
胫骨后倾角°-中轴法:	5°
外平台切骨厚度（mm）:	7.5
内平台切骨厚度（mm）:	3
外平台前后径（mm）:	41.5
平台中部前后径（mm）:	45
内平台前后径（mm）:	49
平台内外径（mm）:	73
股骨远端内侧切骨厚度(mm):	8
股骨远端外侧切骨厚度(mm):	9.5
股骨前方内侧切骨厚度(mm):	0
股骨前方外侧切骨厚度(mm):	4.5
股骨内后髁切骨厚度（mm）:	10.5
股骨外后髁切骨厚度（mm）:	11.5
术前测量股骨尺寸: A□, B□, C□, D□, E☑, F□, G□, H□	
术中测量股骨尺寸: A□, B□, C□, D□, E☑, F□, G□, H□	
术前测量胫骨尺寸: 1号□, 2号□, 3号□, 4号☑, 5号□, 6号□, 7号□, 8号□	
术中测量胫骨尺寸: 1号□, 2号□, 3号□, 4号☑, 5号□, 6号□, 7号□, 8号□	
聚乙烯垫厚度（mm）: 10	
**如果置换髌骨做以下记录**	
髌骨上下极距离: 62.5 (cm)， 内侧头止点距髌骨下支: 1.5 (cm)	
髌骨假体直径(mm):	
髌骨厚度(mm):	21
髌骨假体厚度(mm):	
保留髌骨骨质厚度(mm):	
切除髌骨骨质厚度(mm):	

骨质评估: 优 ✓　中　差

术者：

中国人膝关节数据库

手术日期：2011 年 10 月 13 日，住院号：4628645，“ ”处请打√选择

患者姓名：[redacted] 男 或 女☑： 年龄：66 岁 左膝 或 **右膝**

膝内翻角° 或膝外翻角°：7°

股骨外翻角°：7°

胫骨后倾角°-前缘法：10°

胫骨后倾角°-中轴法：7°

外平台切骨厚度（mm）：10

内平台切骨厚度（mm）：

外平台前后径（mm）：42.5

平台中部前后径（mm）：48.5

内平台前后径（mm）：51.5 （估）

平台内外径（mm）：70 （估）

股骨远端内侧切骨厚度(mm)：7.5

股骨远端外侧切骨厚度(mm)：10.5

股骨前方内侧切骨厚度(mm)：3.5

股骨前方外侧切骨厚度(mm)：9

股骨内后髁切骨厚度（mm）：11

股骨外后髁切骨厚度（mm）：6.5

术前测量股骨尺寸：A□，B□，C□，D□，E☑，F□，G□，H□

术中测量股骨尺寸：A□，B□，C□，D☑，E□，F□，G□，H□

术前测量胫骨尺寸：1号□，2号□，3号□，4号☑，5号□，6号□，7号□，8号□

术中测量胫骨尺寸：1号□，2号□，3号☑，4号□，5号□，6号□，7号□，8号□

聚乙烯垫厚度（mm）：10

如果置换髌骨做以下记录

髌骨上下极距离：42（cm），内侧头止点距髌骨下支：11（cm）

髌骨假体直径(mm)：Ø26

髌骨厚度(mm)：24

髌骨假体厚度(mm)：7.5

保留髌骨骨质厚度(mm)：14.5

切除髌骨骨质厚度(mm)：9.5

骨质评估：好☑ 中 差

[illegible] 13

[illegible]

中国人膝关节数据库

手术日期：2011 年 12 月 13 日，　住院号：4483922，" " 处请打✓选择

患者姓名：[redacted]　男 或 女（✓）：	年龄：71岁　左膝 或 右膝（✓）
膝内翻角° 或膝外翻角°：	10
股骨外翻角°：	8°
胫骨后倾角°-前缘法：	18°
胫骨后倾角°-中轴法：	10°
外平台切骨厚度（mm）：	8.5
内平台切骨厚度（mm）：	2.5
外平台前后径（mm）：	40
平台中部前后径（mm）：	48.5
内平台前后径（mm）：	55
平台内外径（mm）：	75
股骨远端内侧切骨厚度(mm)：	8+2
股骨远端外侧切骨厚度(mm)：	6+2
股骨前方内侧切骨厚度(mm)：	3.5
股骨前方外侧切骨厚度(mm)：	7.5
股骨内后髁切骨厚度（mm）：	17
股骨外后髁切骨厚度（mm）：	15
术前测量股骨尺寸：A□，B□，C□，D□，E□（✓），F□，G□，H□	
术中测量股骨尺寸：A□，B□，C□，D□，E□（✓），F□，G□，H□	
术前测量胫骨尺寸：1号□，2号□，3号□，4号□（✓），5号□，6号□，7号□，8号□	
术中测量胫骨尺寸：1号□，2号□，3号□，4号□（✓），5号□，6号□，7号□，8号□	
聚乙烯垫厚度（mm）：10	

如果置换髌骨做以下记录

髌骨上下极距离：49 (cm)，内侧头止点距髌骨下支：27 (cm)	
髌骨假体直径(mm)：	40.6
髌骨厚度(mm)：	23
髌骨假体厚度(mm)：	7.5
保留髌骨骨质厚度(mm)：	15.5
切除髌骨骨质厚度(mm)：	7.5

[illegible]

床号: 29

术者:

中国人膝关节数据库

手术日期: 2011 年 12 月 14 日, 住院号: 4627598, “ ”处请打✓选择

患者姓名: [redacted] 男 或 女✓: 年龄: 68 岁	
膝内翻角°✓ 或膝外翻角°:	左膝✓ 或 右膝
股骨外翻角°:	3°
胫骨后倾角°-前缘法:	9.3°
胫骨后倾角°-中轴法:	12.6°
外平台切骨厚度(mm):	10°
内平台切骨厚度(mm):	9.5
外平台前后径(mm):	3.5
平台中部前后径(mm):	41
内平台前后径(mm):	42
平台内外径(mm):	50
股骨远端内侧切骨厚度(mm):	75
股骨远端外侧切骨厚度(mm):	8
股骨前方内侧切骨厚度(mm):	9.5
股骨前方外侧切骨厚度(mm):	0
股骨内后髁切骨厚度(mm):	4.5
股骨外后髁切骨厚度(mm):	9.5
	8.5
术前测量股骨尺寸: A□, B□, C□, D□, E☑, F□, G□, H□	
术中测量股骨尺寸: A□, B□, C□, D□, E☑, F□, G□, H□	
术前测量胫骨尺寸: 1号□, 2号□, 3号□, 4号☑, 5号□, 6号□, 7号□, 8号□	
术中测量胫骨尺寸: 1号□, 2号□, 3号□, 4号☑, 5号□, 6号□, 7号□, 8号□	
聚乙烯垫厚度(mm): 10	

如果置换髌骨做以下记录

髌骨上下极距离: 44.5 (cm), 内侧头止点距髌骨下支: 18.5 (cm)	
髌骨假体直径(mm):	42.6
髌骨厚度(mm):	23
髌骨假体厚度(mm):	7.5
保留髌骨骨质厚度(mm):	15.5
切除髌骨骨质厚度(mm):	7.5

骨质评估: 优✓ 中 差

[illegible] N

术后

中国人膝关节数据库

手术日期：2013 年 3月 13日， 住院号：4596837，“ ”处请打✓选择

患者姓名：[redacted] 男 或 女✓： 年龄：79 岁	左膝 或 右膝✓
膝内翻角°✓ 或膝外翻角°：	18.4°
股骨外翻角°：	10.6°
胫骨后倾角°-前缘法：	13.3°
胫骨后倾角°-中轴法：	9.4°
外平台切骨厚度（mm）：	10
内平台切骨厚度（mm）：	0
外平台前后径（mm）：	39
平台中部前后径（mm）：	40
内平台前后径（mm）：	40
平台内外径（mm）：	68
股骨远端内侧切骨厚度(mm)：	5.5 + 2
股骨远端外侧切骨厚度(mm)：	4.5 + 2
股骨前方内侧切骨厚度(mm)：	2.5
股骨前方外侧切骨厚度(mm)：	6
股骨内后髁切骨厚度（mm）：	12.5
股骨外后髁切骨厚度（mm）：	9
术前测量股骨尺寸：A□，B□，C□，D□，E☑，F□，G□，H□	
术中测量股骨尺寸：A□，B□，C□，D□，E☑，F□，G□，H□	
术前测量胫骨尺寸：1号□，2号□，3号□，4号☑，5号□，6号□，7号□，8号□	
术中测量胫骨尺寸：1号□，2号□，3号☑，4号□，5号□，6号□，7号□，8号□	
聚乙烯垫厚度（mm）：10	
如果置换髌骨做以下记录	
髌骨上下极距离：42 (cm)， 内侧头止点距髌骨下支：18 (cm)	
髌骨假体直径(mm)：	Φ26
髌骨厚度(mm)：	22.5
髌骨假体厚度(mm)：	7.5
保留髌骨骨质厚度(mm)：	15
切除髌骨骨质厚度(mm)：	7.5

骨质评估：优 中 差✓

康3.7 术者：

中国人膝关节数据库

手术日期：2013年2月13日，住院号：19087，"" 处请打✓选择

患者姓名：[redacted] 男 或 女✓： 年龄：71岁 左膝✓ 或 右膝	
膝内翻角°✓ 或膝外翻角°：	16°
股骨外翻角°：	3°
胫骨后倾角°-前缘法：	18.6°
胫骨后倾角°-中轴法：	17°
外平台切骨厚度（mm）：	14
内平台切骨厚度（mm）：	8.5
外平台前后径（mm）：	41
平台中部前后径（mm）：	45
内平台前后径（mm）：	49
平台内外径（mm）：	75
股骨远端内侧切骨厚度(mm)：	9
股骨远端外侧切骨厚度(mm)：	8
股骨前方内侧切骨厚度(mm)：	3.5
股骨前方外侧切骨厚度(mm)：	7
股骨内后髁切骨厚度（mm）：	12
股骨外后髁切骨厚度（mm）：	8.5
术前测量股骨尺寸：A□，B□，C□，D□，E☑，F□，G□，H□	
术中测量股骨尺寸：A□，B□，C□，D□，E☑，F□，G□，H□	
术前测量胫骨尺寸：1号□，2号□，3号□，4号☑，5号□，6号□，7号□，8号□	
术中测量胫骨尺寸：1号□，2号□，3号☑，4号□，5号□，6号□，7号□，8号□	
聚乙烯垫厚度（mm）：10	
**如果置换髌骨做以下记录**	
髌骨上下极距离：42（cm），内侧头止点距髌骨下支：14（cm）	
髌骨假体直径(mm)：	
髌骨厚度(mm)：	20.5
髌骨假体厚度(mm)：	
保留髌骨骨质厚度(mm)：	
切除髌骨骨质厚度(mm)：	

骨质评估：优 中 差✓

案号 7 [illegible]

中国人膝关节数据库

手术日期：2013 年 3 月 1 日，住院号：24868，“ ”处请打✓选择

患者姓名：[redacted] 男 或 女✓： 年龄：62 岁 左膝 或 右膝✓	
膝内翻角° 或膝外翻角°✓：	20°
股骨外翻角°：	13°
胫骨后倾角°-前缘法：	10°
胫骨后倾角°-中轴法：	8°
外平台切骨厚度（mm）：	7+2
内平台切骨厚度（mm）：	6.5+2
外平台前后径（mm）：	40
平台中部前后径（mm）：	45
内平台前后径（mm）：	39
平台内外径（mm）：	68
股骨远端内侧切骨厚度(mm)：	10.5
股骨远端外侧切骨厚度(mm)：	6
股骨前方内侧切骨厚度(mm)：	6
股骨前方外侧切骨厚度(mm)：	12.5
股骨内后髁切骨厚度（mm）：	10
股骨外后髁切骨厚度（mm）：	7
术前测量股骨尺寸：A□，B□，C□，D□✓，E□，F□，G□，H□	
术中测量股骨尺寸：A□，B□，C□，D□✓，E□，F□，G□，H□	
术前测量胫骨尺寸：1号□，2号□，3号□✓，4号□，5号□，6号□，7号□，8号□	
术中测量胫骨尺寸：1号□，2号□，3号□✓，4号□，5号□，6号□，7号□，8号□	
聚乙烯垫厚度（mm）：10	
如果置换髌骨做以下记录	
髌骨上下极距离：（cm），内侧头止点距髌骨下支：（cm）	
髌骨假体直径(mm)：	
髌骨厚度(mm)：	
髌骨假体厚度(mm)：	
保留髌骨骨质厚度(mm)：	
切除髌骨骨质厚度(mm)：	

骨质评估： 优 中✓ 差

床号：6　　　　　　　　　　　　　　　　　　　　　　　　　　　术者：

中国人膝关节数据库

手术日期：2013 年 3 月 11 日，　住院号：4686317，"　"处请打✓选择

患者姓名：[redacted]　男 或 女✓：	年龄：61 岁　　左膝✓ 或 右膝
膝内翻角°✓　或膝外翻角°：	19°
股骨外翻角°：	6°
胫骨后倾角°-前缘法：	8°
胫骨后倾角°-中轴法：	6°
外平台切骨厚度（mm）：	9.5
内平台切骨厚度（mm）：	0
外平台前后径（mm）：	43
平台中部前后径（mm）：	44
内平台前后径（mm）：	44
平台内外径（mm）：	72
股骨远端内侧切骨厚度(mm)：	9.5
股骨远端外侧切骨厚度(mm)：	10.5
股骨前方内侧切骨厚度(mm)：	3.5
股骨前方外侧切骨厚度(mm)：	4.5
股骨内后髁切骨厚度（mm）：	9.5
股骨外后髁切骨厚度（mm）：	7.5
术前测量股骨尺寸：A☐，B☐，C☐，D☐，E☑，F☐，G☐，H☐	
术中测量股骨尺寸：A☐，B☐，C☐，D☐，E☑，F☐，G☐，H☐	
术前测量胫骨尺寸：1 号☐，2 号☐，3 号☑，4 号☐，5 号☐，6 号☐，7 号☐，8 号☐	
术中测量胫骨尺寸：1 号☐，2 号☐，3 号☑，4 号☐，5 号☐，6 号☐，7 号☐，8 号☐	
聚乙烯垫厚度（mm）：10	
如果置换髌骨做以下记录	
髌骨上下极距离：29(cm)，内侧头止点距髌骨下支：16（cm）	
髌骨假体直径(mm)：	
髌骨厚度(mm)：	20
髌骨假体厚度(mm)：	
保留髌骨骨质厚度(mm)：	
切除髌骨骨质厚度(mm)：	

骨质评估：优　良✓　差
