# Supplementary figures and images for: Femoral anterior condyle height decreases as the distal anteroposterior size increases in total knee arthroplasty: A comparative study
Source: PLoS One. 2024 Feb 26;19(2):e0297634. doi: 10.1371/journal.pone.0297634 (PMC10896507; doi:10.1371/journal.pone.0297634)

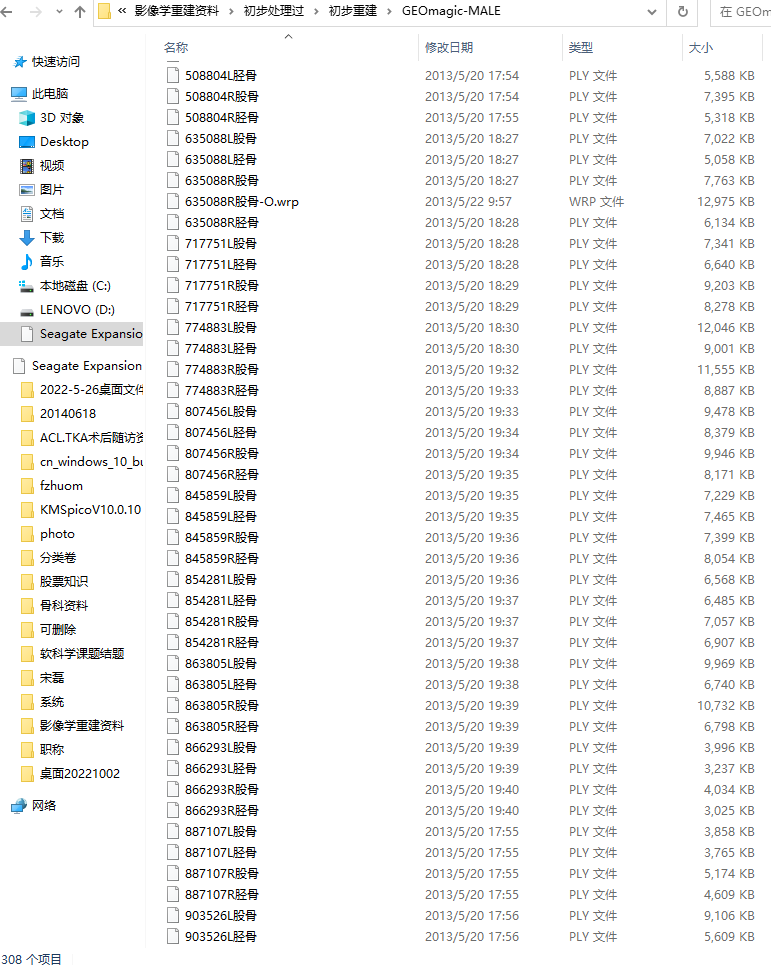

Supplement: S1 File — (ZIP) [file pone.0297634.s001.zip › Supporting Information/Male-geomagic PLY-image data.png]
