# Supplementary material for: Femoral anterior condyle height decreases as the distal anteroposterior size increases in total knee arthroplasty: A comparative study
Source: PLoS One. 2024 Feb 26;19(2):e0297634. doi: 10.1371/journal.pone.0297634 (PMC10896507; doi:10.1371/journal.pone.0297634)
Supplement: Attachment — Submitted filename: Response to Reviewers.docx [file pone.0297634.s002.docx]

Dear Editors and Reviewers:

Thank you very much for your letter and for the reviewers’ careful reading and constructive comments concerning our manuscript entitled “Femoral anterior condyle height decreased as the distal anteroposterior size increasing: contrary to the current designed femoral component” (Manuscript # PONE-D-23-04989). According to editor and reviewers' comments and suggestions, we have revised the manuscript point by point.

We would like to resubmit the revised manuscript to ***PLOS ONE***, and hope it is acceptable for publication in the journal. The detailed changes were tracked in red in the revised manuscript and listed in the following:

Looking forward to hearing from you soon.

Best wishes!

**For editors:**

1. Please ensure that your manuscript meets PLOS ONE's style requirements, including those for file naming. The PLOS ONE style templates can be found at <https://journals.plos.org/plosone/s/file?id=wjVg/PLOSOne_formatting_> sample_main_body.pdf and [https://journals.plos.org/plosone/s/file?id=ba62 /PLOS One_formatting_sample_title_authors_affiliations.pdf](https://journals.plos.org/plosone/s/file?id=ba62%20/PLOS%20One_formatting_sample_title_authors_affiliations.pdf)

**Answer:** Thank you for your good comments. We have edited the manuscript according to PLOS ONE's style requirements.

2. Thank you for stating the following in the Acknowledgments Section of your manuscript:

"This research was funded by Instrument Research Project of the National Natural Science Foundation (No. 81327001). Science and Technology Program of Shaanxi Province (No.2022SF-049)."

We note that you have provided additional information within the Acknowledgements Section that is not currently declared in your Funding Statement. Please note that funding information should not appear in the Acknowledgments section or other areas of your manuscript. We will only publish funding information present in the Funding Statement section of the online submission form.

Please remove any funding-related text from the manuscript and let us know how you would like to update your Funding Statement. Currently, your Funding Statement reads as follows: "The author(s) received no specific funding for this work."

Please include your amended statements within your cover letter; we will change the online submission form on your behalf.

**Answer:** Thank you for your careful reading and suggestions, we have changed funding-related text in revised manuscript and cover letter. This research was funded by Instrument Research Project of the National Natural Science Foundation (No. 81327001), which took part in study design, data collection and analysis, preparation and decision to publish the manuscript; Science and Technology Program of Shaanxi Province (No.2022SF-049),which took part in preparation and revised the manuscript.

3. We note that the grant information you provided in the ‘Funding Information’ and ‘Financial Disclosure’ sections do not match.

When you resubmit, please ensure that you provide the correct grant numbers for the awards you received for your study in the ‘Funding Information’ section.

**Answer:** Thank you very much, we have corrected grant numbers in the ‘Funding Information’ section.

4. Thank you for stating the following financial disclosure:

"The author(s) received no specific funding for this work."

At this time, please address the following queries:

a)   Please clarify the sources of funding (financial or material support) for your study. List the grants or organizations that supported your study, including funding received from your institution.

b)   State what role the funders took in the study. If the funders had no role in your study, please state: “The funders had no role in study design, data collection and analysis, decision to publish, or preparation of the manuscript.”

c)     If any authors received a salary from any of your funders, please state which authors and which funders.

d)    If you did not receive any funding for this study, please state: “The authors received no specific funding for this work.”

Please include your amended statements within your cover letter; we will change the online submission form on your behalf.

**Answer:** Thank you, we have changed funding-related text in revised manuscript and cover letter.

5. In your Data Availability statement, you have not specified where the minimal data set underlying the results described in your manuscript can be found. PLOS defines a study's minimal data set as the underlying data used to reach the conclusions drawn in the manuscript and any additional data required to replicate the reported study findings in their entirety. All PLOS journals require that the minimal data set be made fully available. For more information about our data policy, please see <http://journals.plos.org/plosone/s/data-availability>.

Upon re-submitting your revised manuscript, please upload your study’s minimal underlying data set as either Supporting Information files or to a stable, public repository and include the relevant URLs, DOIs, or accession numbers within your revised cover letter. For a list of acceptable repositories, please see <http://journals.plos.org/plosone/s/data-availability#loc-recommended-repositories>. Any potentially identifying patient information must be fully anonymized.

Important: If there are ethical or legal restrictions to sharing your data publicly, please explain these restrictions in detail. Please see our guidelines for more information on what we consider unacceptable restrictions to publicly sharing data: <http://journals.plos.org/plosone/s/data-availability#loc-unacceptable-data-access-restrictions>. Note that it is not acceptable for the authors to be the sole named individuals responsible for ensuring data access.

We will update your Data Availability statement to reflect the information you provide in your cover letter.

**Answer:** Thank you very much, all relevant data are within the manuscript and its supporting information files. We have provide the data collection process in supporting information, others researchers can replicate our study according the methods in the manuscript. We have described it online submit system. Thank you.

6. We note that you have stated that you will provide repository information for your data at acceptance. Should your manuscript be accepted for publication, we will hold it until you provide the relevant accession numbers or DOIs necessary to access your data. If you wish to make changes to your Data Availability statement, please describe these changes in your cover letter and we will update your Data Availability statement to reflect the information you provide

**Answer:** Thank you for your kindly remind us that we can not provide repository information for our data after acceptance. Because of we are applying for a patent and designing mew femoral prosthesis according to the data now. we have described it online and in the cover letter.

7. PLOS requires an ORCID iD for the corresponding author in Editorial Manager on papers submitted after December 6th, 2016. Please ensure that you have an ORCID iD and that it is validated in Editorial Manager. To do this, go to ‘Update my Information’ (in the upper left-hand corner of the main menu), and click on the Fetch/Validate link next to the ORCID field. This will take you to the ORCID site and allow you to create a new iD or authenticate a pre-existing iD in Editorial Manager. Please see the following video for instructions on linking an ORCID iD to your Editorial Manager account: <https://www.youtube.com/watch?v=_xcclfuvtxQ>.

**Answer:** Thank you，I have updated my information and authenticate ORCID iD.

8. Please include captions for your Supporting Information files at the end of your manuscript, and update any in-text citations to match accordingly. Please see our Supporting Information guidelines for more information: <http://journals.plos.org/plosone/s/supporting-information>.

**Answer:** Thank you very much.

**Additional Editor Comments:**

As you know, the manuscript has a strong title and theme. Which sets the stage for a true revolution of the modern concept of knee prosthesis.

In general, as you can read from the following reports of the reviewers, it emerged that in order to suggest a new prosthetic design, more knowledge on many aspects is needed.

**Answer:** Thank you very much. We have revised the manuscript point by point as fellows according to reviewers’ comments.

**Reviewers' comments:**

Reviewer's Responses to Questions

**Comments to the Author**

1. Is the manuscript technically sound, and do the data support the conclusions?

The manuscript must describe a technically sound piece of scientific research with data that supports the conclusions. Experiments must have been conducted rigorously, with appropriate controls, replication, and sample sizes. The conclusions must be drawn appropriately based on the data presented.

Reviewer #1: Yes

Reviewer #2: Partly

Reviewer #3: Partly

**Answer:** Thank you for your comments. We have amended the whole manuscript carefully in the revised manuscript.

2. Has the statistical analysis been performed appropriately and rigorously?

Reviewer #1: Yes

Reviewer #2: Yes

Reviewer #3: No

**Answer:** Thank you for your comments. We have amended the problems of statistical analysis in the revised manuscript.

3. Have the authors made all data underlying the findings in their manuscript fully available?

The [PLOS Data policy](http://www.plosone.org/static/policies.action#sharing) requires authors to make all data underlying the findings described in their manuscript fully available without restriction, with rare exception (please refer to the Data Availability Statement in the manuscript PDF file). The data should be provided as part of the manuscript or its supporting information, or deposited to a public repository. For example, in addition to summary statistics, the data points behind means, medians and variance measures should be available. If there are restrictions on publicly sharing data—e.g. participant privacy or use of data from a third party—those must be specified.

Reviewer #1: Yes

Reviewer #2: Yes

Reviewer #3: Yes

**Answer:** Thank you very much.

4. Is the manuscript presented in an intelligible fashion and written in standard English?

PLOS ONE does not copyedit accepted manuscripts, so the language in submitted articles must be clear, correct, and unambiguous. Any typographical or grammatical errors should be corrected at revision, so please note any specific errors here.

Reviewer #1: Yes

Reviewer #2: Yes

Reviewer #3: No

**Answer:** Thank you very much. Our manuscript had been edited for English-language problems by Paul Kretchmer, Managing Director, San Francisco Edit.

**5. Review Comments to the Author**

Please use the space provided to explain your answers to the questions above. You may also include additional comments for the author, including concerns about dual publication, research ethics, or publication ethics. (Please upload your review as an attachment if it exceeds 20,000 characters)

**Answer:** Thanks. We have answered the questions above.

**Reviewer #1:**

1.GENERAL COMMENTS

Manuscript topic is current and of interest.

The sample of patients completed the survey was satisfactory.

The English language is ok.

The study contains some anatomical features. Although there are some limitations, I would accept it for publication because it presents some interesting things related to total knee arthroplasty.

**Answer:** Thank you very much for your good praise.

2. INTRODUCTION

The introduction provide the necessary background.

The purpose is clear.

**Answer:** Thank you for your comments.

3. METHODS (Material, patients and methods)

The design of the study is acceptable.

**Answer:** Thank you.

4. RESULTS

ok

**Answer:** Thanks.

5. DISCUSSION

The discussion is ok, although you should state that there are many designs that take into account the dimensions of the femur and that with the newest techniques like robotics the surgeon can calculate the amount of the bone that is necessary to be cut and adjust it to the specific implant design to be used in each patient.

**Answer:** Thank you for your comments.

6. CONCLUSION

ok

**Answer:** Thanks again.

7. ABSTRACT

ok

**Answer:** Thank you.

8. TITLE

The title is ok.

**Answer:** Thanks.

9. REFERENCES

ok

**Answer:** Thank you very much.

10. TABLES

ok

**Answer:** Thank you again.

**Reviewer #2:**

Dear authors, i had the opportunity of reviewing your work, and i hope that my comments help strengthening your manuscript. You disclosed that you received no funds, however, later on you acknowledge your funding agency, please correct accordingly.

**Answer:** Thank you for your careful reading and suggestions, we have changed funding-related text in revised files.

TITLE: please indicate in the title that this was a comparative study.

**Answer:** Thank you. We have revised the title as “Femoral anterior condyle height decreases as the distal anteroposterior size increases in total knee arthroplasty: A comparative study ” in revised manuscript(line 5-6).

ABSTRACT: please made it clear that the measurements were clinical in the TKA cases and radiographic in the CT scans.

**Answer:** Thank you, we have described the measurements were undergoing TKA intraoperative and computed tomography scans in revised manuscript (line 45-46).

INTRODUCTION:

-references 10,11 in line 82 are old, please update

**Answer:** Thank you for your suggestion, we have updated the references 10,11 in revised manuscript(line 297-299).

10. Li P, Tsai TY, Li JS, Wang S, Zhang Y, Kwon YM, et al. Gender analysis of the anterior femoral condyle geometry of the knee. Knee. 2014; 21(2): 529-533.

11. [Gillespie RJ](http://www.ncbi.nlm.nih.gov/pubmed?term=Gillespie%20RJ%5BAuthor%5D&cauthor=true&cauthor_uid=21357958), [Levine A](http://www.ncbi.nlm.nih.gov/pubmed?term=Levine%20A%5BAuthor%5D&cauthor=true&cauthor_uid=21357958), [Fitzgerald SJ](http://www.ncbi.nlm.nih.gov/pubmed?term=Fitzgerald%20SJ%5BAuthor%5D&cauthor=true&cauthor_uid=21357958), [Kolaczko J](http://www.ncbi.nlm.nih.gov/pubmed?term=Kolaczko%20J%5BAuthor%5D&cauthor=true&cauthor_uid=21357958), [DeMaio M](http://www.ncbi.nlm.nih.gov/pubmed?term=DeMaio%20M%5BAuthor%5D&cauthor=true&cauthor_uid=21357958), [Marcus RE](http://www.ncbi.nlm.nih.gov/pubmed?term=Marcus%20RE%5BAuthor%5D&cauthor=true&cauthor_uid=21357958), [et](http://www.ncbi.nlm.nih.gov/pubmed?term=Cooperman%20DR%5BAuthor%5D&cauthor=true&cauthor_uid=21357958) al. Gender differences in the anatomy of the distal femur. [J Bone Joint Surg Br.](http://www.ncbi.nlm.nih.gov/pubmed/21357958) 2011; 93(3):357-63.

lines 85 to 87: seems out of context, please correct or remove

**Answer:** Thank you for your suggestion, we have corrected these sentences in the revised manuscript(line 82-85).

please rephrase the aim of the study and report it into primary objective and secondary objectives, as the authors through the methods and results reported on may differences not only the assessment of the anterior condylar height.

**Answer:** Thank you for your good suggestion. We have added secondary objectives that whether gender differences exist in anterior condyle height in the revised manuscript(line43-44).

METHODS: - why the data was collected a long time ago (till 2015), it is almost 8 years, where major changes were made in the design of the implants, furthermore, as long as this was a historical group, why the authors did not report any data regarding the development of PFJ complications which is the main concerned point with their study.

**Answer:** This study mainly clarified the anatomy features we found that the femoral anterior condyle height decreases with the increase of the distal femur dimension, which is different from the prosthesis currently used (femoral anterior flange increases with the prosthesis size increased). During that period, we didn't publish a paper because we applied for a patent and designed mew femoral prosthesis according to the data. In clinic, some patients have PFJ-related complications after TKA, including prosthesis overfilling, which is partly due to that the anterior flange thickness of prosthesis is greater than that of anterior condyle resection thickness. I hope I can answer your question. Thank you.

-the authors reported using one implant for all of their cases, i was wondering why they did not report a comparison between the sizes of the femoral component anterior flange and compare it against their population. Furthermore, as long as they already know the femoral component sizes placed in each patient, they could have tested theses sizes against the measured resected anterior femoral cut.

**Answer:** Thanks, the question you raised is very good, and we have neglected this aspect. Later, we will collect relevant data and make a summary. This paper mainly wants to explain the anatomy features we found that the femoral anterior condyle height decreases with the increase of the distal femur dimension, which is different from the prosthesis currently used (femoral anterior flange increases with the prosthesis size increased).

-The authors did not report any inclusion or exclusion criteria, did they include all forms and magnitudes of deformities? patients with different pathologies such as RA, or patients with posttraumatic OA where the anatomy could be distorted?

**Answer:** Thank you. The criteria for inclusion and exclusion of cases are very important. We choose primary OA subjects for this study. Patients were excluded if they had a history of femur fracture or congenital anomaly or if the bone loss or degradation was so serious that augmentation was required or if the knee had a varus or valgus deformity of >15°. We have described it in the revised manuscript(line 95-99).

What was the exact technique for performing the TKA especially the rotation which could affect the anterior femoral condyles cut thickness?

**Answer:** Generally, The femoral external rotation 3°relative to the posterior condylar axis for the distal femoral cut, or paralleling the transepicondylar axis or perpendicular to Whitesides’ line to ensure the flexion gap balance. We have described it in the revised manuscript(line 102-104).

RESULTS:

The authors concentrated their comparisons on the gender differences, supposing that the CT group were a normal group, why the authors did not compare both groups to detect if there was a disturbance according to the pathological process leading to performing TKA.

**Answer:** Thank you, it is a good question. The pathology of TKA patients may have some influences on the anatomy of knee joint. The cartilage of TKA patients was partly worn or even disappeared completely, and there was no cartilage exit in CT image. In addition, the present study mainly to elucidate the anatomical features that anterior condyle height decreased as the increase of the distal femur. CT images and TKA intraoperative measurements verify the results for each other.

Could the differences between bone cuts thickness between males and females be attributed to the fact that the male skeleton is larger than the female skeleton?

**Answer:** Many literatures have previously reported that gender differences of knee anatomy were the result of a smaller skeleton in females. Femoral anterior condylar cuts thickness between males and females partly attributed to the fact that the male skeleton is larger than the female skeleton. In addition, we found significant gender differences of anterior condylar resection thickness.

The authors stated that "the dimensions of the anterior condylar height were highly variable, regardless of gender.", how could this be solved by changing the implants design? could a custom made implant for each patient becomes the solution?

**Answer:** Our results showed that there is a negative correlation between the femoral anterior condyle height and the distal femur size. But the individual is highly variable. Custom-made prostheses are the best solution, and many researchers and companies are now working on personalized design and 3D printing. our research group also has been working in this area, and we believe it can be realized in the near future.

DISCUSSION:

-Please indicate some possible solutions in view of your results.

**Answer:** Thanks, according to our results, in order to better fit the femoral anterior condyles, when designed femoral prosthesis, the large femoral implant should have a thinner anterior flange, and the small femoral implant should have a thicker anterior flange. Theoretically, femoral components designed like this should more closely approximate the thickness of the original resected anterior condyle.

Reviewer #3: Thank you for giving me the opportunity to review this manuscript. The authors describe differences in the dimensions regarding distal femur morphology of patients undergoing TKA and participants with "healthy" knees.

Although I believe the manuscript would have some interest for the reader, I believe it is not ready for publication in the current form since there are significant methodological and language issues which cannot be ignored.

**Answer:** Thank you very much. We have edited the language issues of whole manuscript by a native speaker.

General comment:

I've never read or heard the term "condyle" regarding the anterior part of the distal femur. Usually the terms "anterior cortex" etc. are used.

**Answer:** Thank you for your question. The term "condyle" regarding the anterior part of the distal femur usually used the terms "anterior cortex" etc. But some authors use it too, such as:

Fehring TK, Odum SM, Hughes J, [Springer BD](http://www.ncbi.nlm.nih.gov/pubmed?term=Springer%20BD%5BAuthor%5D&cauthor=true&cauthor_uid=19797567), [Beaver WB Jr](http://www.ncbi.nlm.nih.gov/pubmed?term=Beaver%20WB%20Jr%5BAuthor%5D&cauthor=true&cauthor_uid=19797567). Differences between the sexes in the anatomy of the anterior condyle of the knee. J Bone Joint Surg Am. 2009; 91-A: 2335-2341.

Ishitani K, Ito T, Hatada R, Kuriyama S, Nakamura S, Ito H, Matsuda S. High and varied anterior condyle of the distal femur is associated with limited flexion in varus knee osteoarthritis. Cartilage. 2021;13(1suppl):1487S-1493S.

If you don't think it's appropriate, later we will change it. Thanks again.

Major issues:

There are many grammatical errors throughout the whole manuscript, which in some parts makes the reading/understanding of the text almost impossible. There is an urgent need for editing by a native speaker. Otherwise I do not see a chance for publication.

**Answer:** Thank you for your comments. Our manuscript had been edited for English-language problems by a native speaker, Paul Kretchmer, Managing Director, San Francisco Edit.

According to the findings of this study. There must be a high rate of patients with anterior overstuffing which should be visible in an impairment of clinical function or patient reported outcome measurements. It should be considered to include clinical data. Additionally, overstuffing should be measurable in postop. radiological evaluation. It should also be considered to include post-TKA CT and correlate the restoration of the anterior cortex by the anterior flange.

**Answer:** Thanks, the question you raised is very good. According to our results, there must be a high rate of patients with anterior overstuffing in bigger knee or understuffing in smaller knee, which finally results in function impairment in clinical, such as anterior knee pain, limiting postoperative knee range of motion, etc. These could be evaluated by radiological method after TKA. However, the mainly purpose in this paper was to clarity the anatomy features that femoral anterior condyle height decreases as the femoral AP size increases. The clinical and radiological evaluation requires further study. Later, we will collect relevant clinical and radiological data, and make a summary. I hope I can answer your question. Thank you again.

Methods: Why do the authors compare healthy and degenerate knees? The implications of the study will mainly influence patients undergoing TKA. Additionally, patients undergoing ACL-reconstruction do not necessarily have a "healthy" knee anatomy.

**Answer:** Thank you very much, it is a good question. The present study mainly to elucidate the anatomical features of femoral anterior condyle height decreased as femoral AP size increases. CT images and TKA intraoperative measurements verify the results for each other. In addition, we selected ACL reconstruction patients with normal knee anatomy, we have described it in revised manuscript(line 123-125).

The authors need to deliver more details on the intraoperative evaluations: It is hard to define "flush" when it comes to the anterior cut of the distal femur. What was the exact definition in your study?

**Answer:** Thank you very much. Anterior referencing instrumentation was used to cut the anterior condyle. The anterior condylar heights were measured intraoperatively only for patients whose anterior condylar cut line flushed with the anterior cortical surface of the distal femoral shaft. We Illustrate it with a legend (fig 1a) in revised manuscript (line 102-107).

**Answer:**

Were varus and valgus deformity included?

**Answer:** Thank you. The criteria for inclusion and exclusion of cases are very important. Patients were excluded if they had a history of femur fracture or congenital anomaly or if the bone loss or degradation was so serious that augmentation was required or if the knee had a varus or valgus deformity of >15°. We have described it in the revised manuscript (line 95-99).

How much external rotation was used for the distal femoral cut?

**Answer:** Thank you. Generally, The femoral external rotation 3°relative to the posterior condylar axis for the distal femoral cut, or paralleling the transepicondylar axis or perpendicular to Whitesides’ line to ensure the flexion gap balance. We have described it in the revised manuscript (line 102-105).

Results:

Can the authors explain the different results from CT and intraop.?

**Answer:** Thank you, it is a good question. The pathology of TKA patients have some influences on the anatomy of knee. The cartilage thickness of TKA cases was partly worn or even disappeared completely, and there was no cartilage exit in CT image. So we didn't compare their differences. In addition, the present study mainly to elucidate the anatomical features of femoral anterior condyle height decreased as femoral AP size increases. CT images and TKA intraoperative measurements verify the results for each other.

Line 180 and following: I cannot find the calculations for the correlations between ALCH and LAP and AMCH and MAP respectively. Correlations should be calculated (e.g. Spearman) to display whether these findings show a significance. The method should then also be named in the M+M section.

Answer: Thank you, we apply linear regression analysis to determine correlations between ALCH and LAP, AMCH and MAP dimensions. We have described it in the revised manuscript (line 154-155).

Discussion:

Line 197-206: The authors report on studies which show results in contradiction to their findings and which support the rationale of contemporary TKA designs --> the bigger the femoral component size the bigger the anterior flange. However, the authors do not elucidate why these differences in their study are explainable.

**Answer:**

Some studies have reported that the anterior condylar height have a positive correlation with the length of the femur or femoral ML size. There has not been a study that analyzed the relationship between anterior condyle height and distal femoral AP size. Our study found that the femoral anterior condyle height decreased with the distal femur size increase, which contrary to the prosthesis currently used. This is the anatomical feature of femoral anterior condyle that we found.

6. PLOS authors have the option to publish the peer review history of their article ([what does this mean?](https://journals.plos.org/plosone/s/editorial-and-peer-review-process#loc-peer-review-history)). If published, this will include your full peer review and any attached files. If you choose “no”, your identity will remain anonymous but your review may still be made public.

Do you want your identity to be public for this peer review? For information about this choice, including consent withdrawal, please see our [Privacy Policy](https://www.plos.org/privacy-policy).

Reviewer #1: No

Reviewer #2: No

Reviewer #3: No

**Answer:** Thank you.

While revising your submission, please upload your figure files to the Preflight Analysis and Conversion Engine (PACE) digital diagnostic tool, <https://pacev2.apexcovantage.com/>. PACE helps ensure that figures meet PLOS requirements. To use PACE, you must first register as a user. Registration is free. Then, login and navigate to the UPLOAD tab, where you will find detailed instructions on how to use the tool. If you encounter any issues or have any questions when using PACE, please email PLOS at [figures@plos.org](mailto:figures@plos.org). Please note that Supporting Information files do not need this step.

**Answer:** Thank you we have deal the figures with PACE digital diagnostic tool and uploaded them to the system again.
